# Intrapulmonary vaccination with delta-inulin adjuvant stimulates non-polarised chemotactic signalling and diverse cellular interaction

**DOI:** 10.1038/s41385-021-00379-6

**Published:** 2021-02-04

**Authors:** Kia C. Ferrell, Erica L. Stewart, Claudio Counoupas, Thomas M. Ashhurst, Warwick J. Britton, Nikolai Petrovsky, James A. Triccas

**Affiliations:** 1 Discipline of Infectious Diseases and Immunology, School of Medical Sciences, Faculty of Medicine and Health, The University of Sydney, Camperdown, NSW Australia; 2 Tuberculosis Research Program, Centenary Institute, The University of Sydney, Camperdown, NSW Australia; 3 Vaxine Pty Ltd, 11 Walkley Avenue, Warradale and Flinders University, Adelaide, Australia; 4 Sydney Cytometry Core Research Facility, Centenary Institute and The University of Sydney, Camperdown, NSW Australia; 5 Marie Bashir Institute for Infectious Diseases and Biosecurity, The University of Sydney, Camperdown, NSW Australia; 6 Department of Clinical Immunology, Royal Prince Alfred Hospital, Camperdown, NSW Australia

## Abstract

There is an urgent need for novel vaccination strategies to combat respiratory pathogens. Mucosal vaccine delivery is an attractive option as it directly targets the site of infection; however, preclinical development has been hindered by a lack of suitable mucosal adjuvants and a limited understanding of their immune effects in the lung environment. Herein, we define the early immune events following the intrapulmonary delivery of a vaccine incorporating the adjuvant delta-inulin. Analysis of the early inflammatory response showed vaccine-induced innate cell recruitment to lungs and local lymph nodes (LN) was transient and non-polarised, correlating with an increase in pulmonary chemotactic factors. Use of fluorescently labelled adjuvant revealed widespread tissue dissemination of adjuvant particles, coupled with broad cellular uptake and transit to the lung-draining LN by a range of innate immune cells. Mass cytometric analysis revealed extensive phenotypic changes in innate and adaptive cell subsets induced by vaccination; this included identification of unconventional lymphocytes such as γδ-T cells and MAIT cells that increased following vaccination and displayed an activated phenotype. This study details a comprehensive view of the immune response to intrapulmonary adjuvant administration and provides pre-clinical evidence to support delta-inulin as a suitable adjuvant for pulmonary vaccines.

## Introduction

There is an urgent need for novel adjuvants suitable for pulmonary delivery. Respiratory pathogens are responsible for some of the most significant diseases of our time, including tuberculosis (TB), responsible for the most deaths per year by a single pathogen, and more recently the COVID-19 pandemic.^1^ Whilst parenteral administration is sufficient for vaccine protection against some diseases, the established vaccine strategies provide insufficient protection for pathogens such as *Mycobacterium tuberculosis*.^2^ Mucosal vaccine delivery, including either intranasal or intrapulmonary administration, has been identified as a promising alternative to parenteral vaccination for TB vaccine candidates, potentially through more efficient resident memory T cell generation.^3,4^ Given the clinical success of the intranasal FluMist vaccine, mucosal vaccines may prove more feasible for clinical translation than other modes of administration.^5^

Subunit vaccines are ideal candidates for mucosal delivery because of their predictability and safety, depending on the selection of adjuvant for vaccine formulation. While there are oral and intranasal vaccines approved for clinical use, none are currently approved for intrapulmonary delivery.^6^ Hindering progress in this field is limited understanding of the adjuvant properties required for vaccine efficacy and safety when formulations are delivered to the pulmonary mucosa.^6,7^ The focus of intrapulmonary and intranasal adjuvant development has been primarily on the identification of adjuvant delivery systems that are demonstrably safe and are sufficiently immunogenic to overcome the tolerogenic response in the pulmonary mucosa, and in the case of intrapulmonary delivery specifically, maintaining their structure and function after nebulisation or spray-drying.^6,8,9^

Advax (delta-inulin) is a particulate adjuvant currently being studied in numerous vaccine platforms, including against Hepatitis B, pandemic influenza, bee-sting allergy and more recently *M. tuberculosis* in a pulmonary vaccine candidate.^10–13^ Advax is also a component of the SARS-CoV-2 vaccine candidate COVAX19, currently in phase I clinical trials (Clinical Trials Identifier NCT04453852). The safety of Advax has been demonstrated in multiple clinical trials and the low reactogenicity observed after parenteral administration makes it a promising candidate for pulmonary delivery.^14,15^ In recent pre-clinical studies, it has been demonstrated that Advax as a mucosal adjuvant generates systemic and mucosal antibody responses and enhances T cell mediated responses.^13,16^

There is increasing interest in delineating the exact mechanism of adjuvants in clinical use, for example by studying innate immune cell interactions with fluorescently-conjugated adjuvants.^17^ While the dynamics of leucocyte responses to parenteral adjuvant administration have been characterised, there are few detailed analyses of innate immune cell recruitment, uptake and activation induced by pulmonary adjuvant candidates and greater understanding in this area is still required.^18–20^ In the present study, fluorescein-conjugated Advax was administered intratracheally (i.t.) either alone or with the CysVac2 fusion protein, a TB vaccine candidate that is highly immunogenic and protective in pre-clinical studies and generates enhanced protection when administered as a pulmonary vaccine.^13,21,22^ Using confocal microscopy and flow cytometry, we performed a comprehensive analysis of innate cell recruitment and adjuvant interaction at different time points after vaccination. Examination of the local cytokine and chemokine environment and analysis of cellular marker expression by mass cytometry provided insight into vaccine-induced changes in immune cell activation. Our data suggest that Advax is a highly chemotactic adjuvant when administered into the lungs and interacts with a broad range of innate immune cells. Furthermore, despite its retention in pulmonary immune cells, i.t. vaccination appears to induce a transient inflammatory response that resolves over time.

## Results

### Advax colocalizes with CysVac2 protein and is distributed throughout the lung tissue following intratracheal administration

**Fig. 1:**
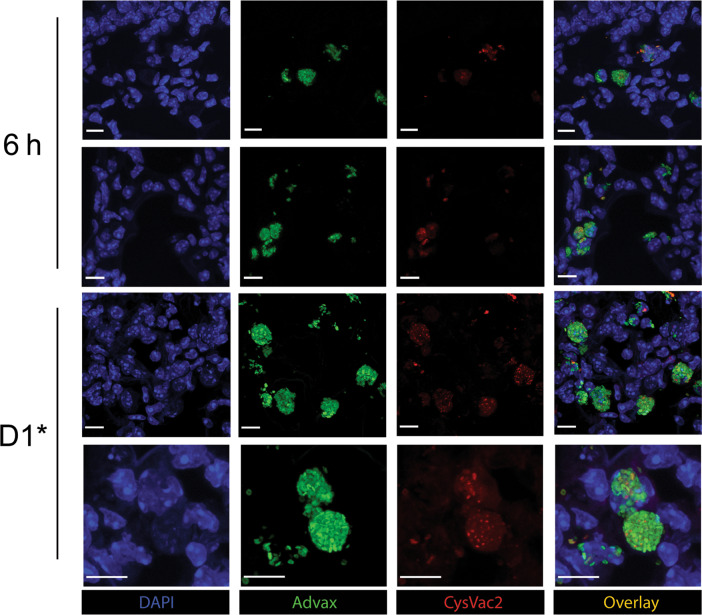
Advax adjuvant and CysVac2 antigen colocalize after intratracheal administration. C57BL/6 mice were i.t. immunised with one or two doses of 3 µg CysVac2-AF647/1 mg Advax-FITC and lung lobes imaged by confocal microscopy. Colocalization of CysVac2-AF647/Advax-FITC was imaged at 6 h post immunisation or 1 day post boost immunisation. Bar lengths correspond to 10 µm.

**Fig. 2:**
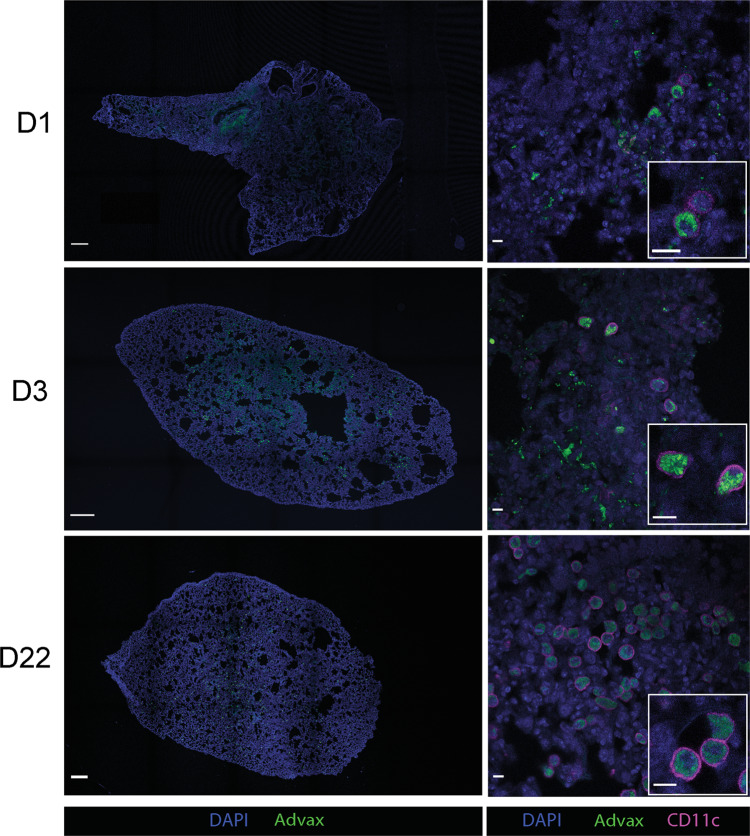
Advax adjuvant and CysVac2 antigen are uniformly distributed throughout the lung after intratracheal administration. C57BL/6 mice were i.t. immunised with one dose of CysVac2/Advax-FITC and lung lobes imaged by confocal microscopy. Whole lobe images of mice vaccinated with CysVac2/Advax-FITC harvested at 1, 3 and 22 days post-vaccination, stained with DAPI (blue) and anti-CD11c-AF647 (magenta). Bar lengths in whole lobe images correspond to 500 μm, in second column images correspond to 10 μm, and inset images to 5 μm.

We sought to visualise localisation of adjuvant and antigen within the lung following intratracheal vaccination using fluorescently conjugated vaccine components. When the CysVac2 fusion protein conjugated to the fluorescent dye AF647 was delivered intratracheally (i.t.) with fluorescein-conjugated Advax, colocalization of adjuvant and antigen was observed both in the extracellular lung tissue and within cells at both 6 h and 1 day post-vaccination or boost (Fig. 1). Similar patterns of colocalization were also observed in murine bone marrow-derived dendritic cells co-cultured with both fluorescent vaccine components (Fig. S1). To investigate further the distribution of Advax adjuvant throughout lung tissue, whole lobe sections were stained with anti-CD11c and imaged after intrapulmonary administration (Fig. 2). Advax adjuvant was dispersed throughout the entire lobe at 1 day post-vaccination and had been partially internalised by CD11c^+^ cells at this time point. The distribution of internalised and extracellular Advax appeared to change over time. There was a noticeable shift towards more intracellular Advax particles up to 22 days following vaccination. These data indicate that CysVac2/Advax appears to be co-distributed in the lungs after pulmonary administration and the vaccine is readily disseminated throughout lung tissue where it is internalised by CD11c^+^ cells.

### Intrapulmonary vaccination with CysVac2/Advax transiently increases diverse immune subsets in the lung

**Fig. 3:**
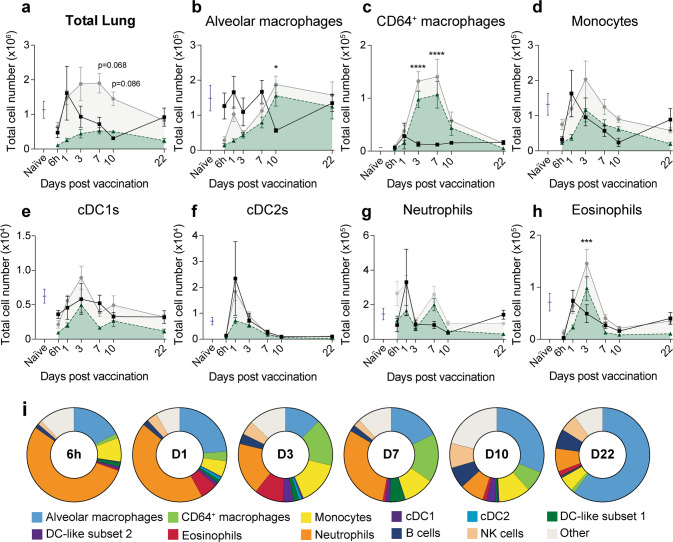
Pulmonary Advax/CysVac2 vaccination induces recruitment of innate subsets to the lung. C57BL/6 mice were left unvaccinated (naïve) or administered CysVac2-AF647/Advax-FITC or PBS control by the i.t. route and 6 h, 1 d, 3 d, 7 d, 10 d and 22 d after vaccination lungs and mediastinal lymph nodes were collected for flow cytometric analysis of innate immune subsets. **a**–**h** Total lung cell number of innate subsets in naïve (blue), PBS (black) and CysVac2/Advax (grey) vaccinated mice, showing the total number of CysVac2/Advax cells within each subset that are Advax-FITC + (shaded green). **i** Proportional distribution of total Advax-FITC present in pulmonary cell subsets identified by flow cytometry following i.t. vaccination with fluorescent CysVac2/Advax; values are determined from the same experiment as **a**–**h**. For **a**–**i**, each value represents the mean ± SEM of *n* = 3–4 per group, and are representative of two independent experiments. *p < 0.05, Two-way ANOVA with Sidak’s post-hoc test.

To identify the kinetics of immune cell recruitment, flow cytometric analysis of immune populations in the lung and mediastinal lymph node (mLN) was performed. Pulmonary vaccination with either PBS or CysVac2/Advax caused an influx of cells at 24 h post administration (Fig. 3a). While by day 3 the lung cell number of PBS-treated animals returned to that of naïve, the cell number in CysVac2/Advax vaccinated groups remained elevated compared to PBS until day 7, only returning to baseline at day 22 post administration (Fig. 3a). We identified immune cells recruited to the lungs using a 13 colour flow cytometry panel and gating strategy (Figure S2) adapted from Misharin et al.^23^ At 6 h post-vaccination, the cellular infiltrate in CysVac2/Advax lungs consisted primarily of neutrophils (Fig. 3g). Eosinophils and conventional dendritic cell subset 1 (cDC1) cells peaked 3 days following CysVac2/Advax vaccination, as did monocytes (Fig. 3d, e, h). The PBS group also experienced variable recruitment of monocytes, cDC2 cells and neutrophils at 24 h post vaccination, all of which returned to numbers comparable to naïve by 3 days. In CysVac2/Advax vaccinated animals at 7 days post-vaccination, CD64^+^ macrophages and neutrophils showed increased accumulation in the lung (Fig. 3c, g), but all subsets returned to that of naïve animals by day 22. Overall, i.t. administration of CysVac2/Advax induced an increase in a diverse range of innate cell subsets in the lung.

The measurement of adjuvant uptake by specific cell subsets was examined by the use of fluorescein-conjugated Advax particles. All immune subsets showed a degree of association with the adjuvant at any given time point, with fluctuations in the proportion of adjuvant-associated cells within each subset across the time course measured (Fig. 3b–h, green shading indicating proportion of cell subset in CysVac2/Advax lungs that is fluorescein positive). When examining total cells of the lung, at 6 h post-vaccination the proportion of Advax-associated cells was ~10%, however, by day 3 this proportion increased to 20% of all cells. Whilst the total cell number within vaccinated lungs transiently increased and returned to naïve levels by 22 days, the number of Advax positive cells stabilised after 3 days and did not subside over the time course measured. Proportionally, the major subsets responsible for Advax uptake in early timepoints (6 h and day 1) were neutrophils and alveolar macrophages, with neutrophils making up 50% of all Advax positive cells in the lungs (Fig. 3i). At days 3, 7 and 10, cell subsets associated with Advax broadened to include monocytes and CD64^+^ macrophages as well as DC-like cells, defined by their expression of CD11c and MHCII (Fig. S2). By day 22, the majority of Advax-associated cells were alveolar macrophages. In their entirety, these data characterise the diversity of immune cell recruitment and wide range of cellular interactions with the Advax adjuvant in the pulmonary environment.

**Fig. 4:**
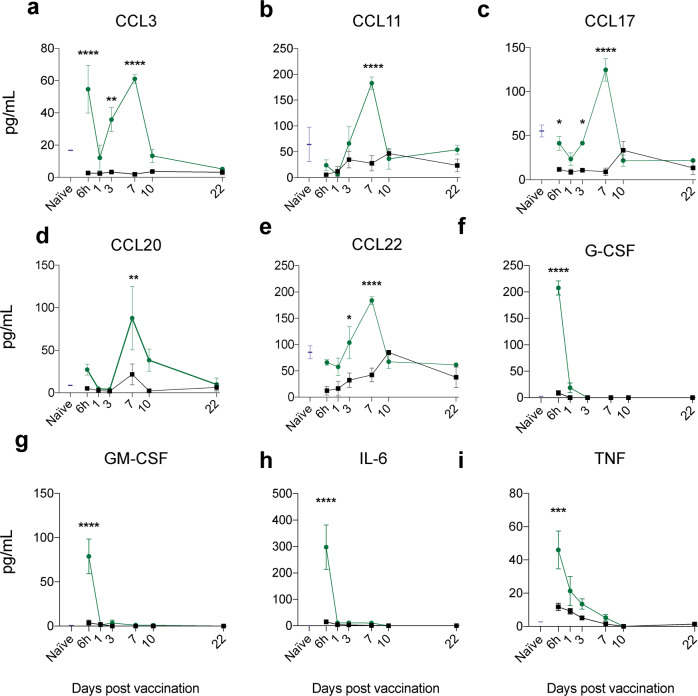
Pulmonary Advax/CysVac2 vaccination induces changes in cytokine and chemokine concentrations in the lung. C57BL/6 mice were administered CysVac2-AF647/Advax-FITC or PBS by i.t. route or left unvaccinated (naïve) and 6 h, 1 d, 3 d, 7 d, 10 d and 22 d after vaccination lungs and mediastinal lymph nodes were collected for flow cytometric analysis. Supernatants of lung single cell suspensions were collected for cytokine and chemokine analysis. **a**–**i** Cytokine and chemokine concentrations measured in lung cell suspension supernatant in naïve (blue), PBS (black) and CysVac2/Advax vaccinated (green) mice following vaccination. Each value represents the mean ±  SEM of *n* = 3–4 per group, and are representative of two independent experiments. Statistical analysis on difference between PBS and CysVac2/Advax mice was analysed using two-way ANOVA with Sidak’s post-hoc test, *p < 0.05.

To examine vaccine and adjuvant-induced changes in the inflammatory milieu of the lung environment, chemokine and cytokine concentrations were determined (Fig. 4). An initial burst of activity was observed in G-CSF, GM-CSF, TNF and IL-6 concentrations at 6 h post-vaccination with CysVac2/Advax which was not observed in the PBS group (Fig. 4f–i). G-CSF, GM-CSF and IL-6 returned to naïve levels by day 1, and TNF was comparable to naïve at day 7. In contrast, the chemokines measured showed a near uniform peak in concentration at 7 days post-vaccination (Fig. 4a–e). Macrophage and granulocyte chemotactic and activation factors such as CCL3 and CCL11, as well as CCL20 and CCL22, responsible for mononuclear cell recruitment, were all induced by CysVac2/Advax administration and not PBS alone (Fig. 4a–e). CCL3 (Fig. 4a) was also observed at levels significantly higher than PBS at 6 h post vaccination, in a similar kinetic to the cytokines previously mentioned. Furthermore, CCL17 and CCL22, both associated with naïve T cell recruitment, were also elevated at 7 days when compared to PBS vaccinated animals (Fig. 4c, e). All the chemokines measured in the lungs of CysVac2/Advax vaccinated mice returned to naïve concentrations by 10 days. Taken together, these data indicate that pulmonary CysVac2/Advax administration stimulates an early cytokine burst that quickly subsides, and is followed by a more substantial rise in chemotactic factors potentially responsible for stimulating the mobilisation of a broad variety of immune subsets to the lung.

### Innate immune cells traffic to the lung-draining lymph node following immunisation with CysVac2/Advax

**Fig. 5:**
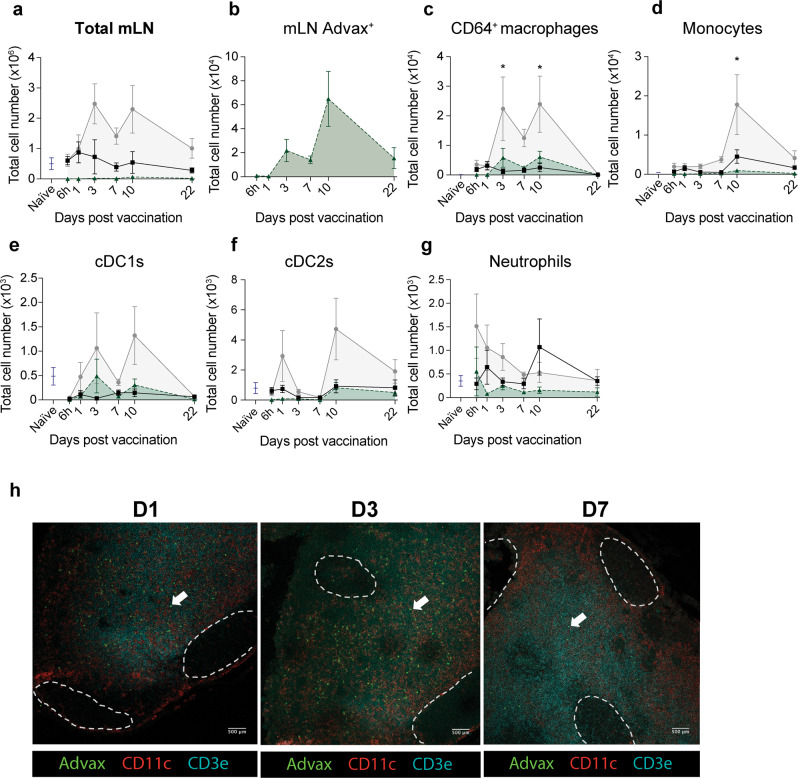
Intrapulmonary Advax/CysVac2 vaccination induces changes in myeloid populations in mediastinal lymph nodes. C57BL/6 mice were left naïve or administered CysVac2-AF647/Advax-FITC or PBS control by i.t. route and 6 h, 1 d, 3 d, 7 d, 10 d and 22 d after vaccination, lungs and mediastinal lymph nodes (mLN) were collected for flow cytometric analysis of innate immune subsets. **a**–**g** Total cell number of innate subsets in naïve (blue), PBS (black) and CysVac2/Advax (grey) vaccinated mice, showing the total number of Advax-FITC positive cells (shaded green) in the mLN. Each value represents the mean ± SEM of *n* = 3–4 per group, representative of two independent experiments. *p < 0.05, Two-way ANOVA with Sidak’s post-hoc test. **h** Mediastinal LN of C57Bl/6 mice vaccinated i.t. with CysVac2/Advax 1 d, 3 d and 7 d after vaccination were snap frozen, sectioned and stained for confocal microscopy with anti-CD3 (cyan) and anti-CD11c (red). White lines indicate B cell follicles; arrows indicate Advax-associated cells.

The stimulation of chemotaxis to the lungs by pulmonary Advax administration was coupled with a marked increase in the cell number of the mLN that was not observed in the PBS control group (Fig. 5a). The total lymph node cell number of the CysVac2/Advax group peaked at 3 and 10 days post-vaccination, with the majority of Advax-associated cells observed at 10 days (Fig. 5b). The earliest arriving cells at the lung-draining lymph node were neutrophils at 6 h, followed by cDC2 cells at 1 day post-vaccination (Fig. 5f, g). At 3 days post-vaccination, Advax-associated CD64^+^ macrophages and cDC1 cells arrived at the lung-draining lymph node, with a secondary peak along with cDC2 cells and monocytes at 10 days post-vaccination (Fig. 5c–f). The anatomical distribution of Advax in the mLN was also determined using confocal microscopy (Fig. 5h). By delineating the T cell zones and B cell follicles using anti-CD3 and anti-CD11c it was observed that Advax particles localised only in the T cell zone of the lymph node and were not present in B cell follicles, with Advax particles visible up to 7 days post-vaccination (Fig. 5h). These data indicate the chemotactic signals induced by intrapulmonary Advax administration stimulate the recruitment of a broad variety of immune cells to the lung, where they interact and uptake vaccine components before migrating to T cell zones of the mediastinal lymph node.

### Pulmonary CysVac2/Advax induces the recruitment and activation of unconventional innate immune cells in the lung and mediastinal lymph node

**Fig. 6:**
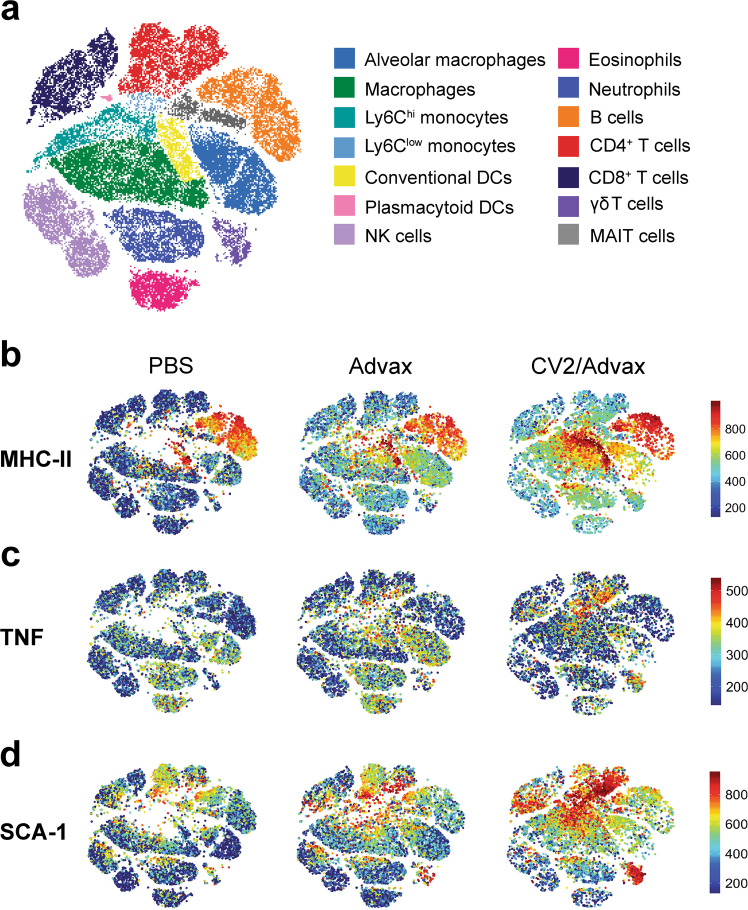
Intrapulmonary administration with CysVac2/Advax induces the recruitment and activation of a diverse range of innate cell types. C57BL/6 mice were vaccinated i.t. with PBS, Advax-FITC or CysVac2/Advax-FITC and lungs were harvested for mass cytometric analysis 7 days later. **a** t-SNE representation was generated using FlowJo software by clustering on phenotypic markers identified in Supplementary table 1 and subset identity was confirmed using the manual gating strategy detailed in Fig. S5. **b–d** Representative t-SNE graphs showing the fluorescence intensity expression (scale bars indicating range of fluorescence intensity) of MHC-II, TNF and SCA-1 in the lungs of vaccinated animals were generated using R software and are representative of *n* = 3 or 4 mice per experiment and two independent experiments.

**Fig. 7:**
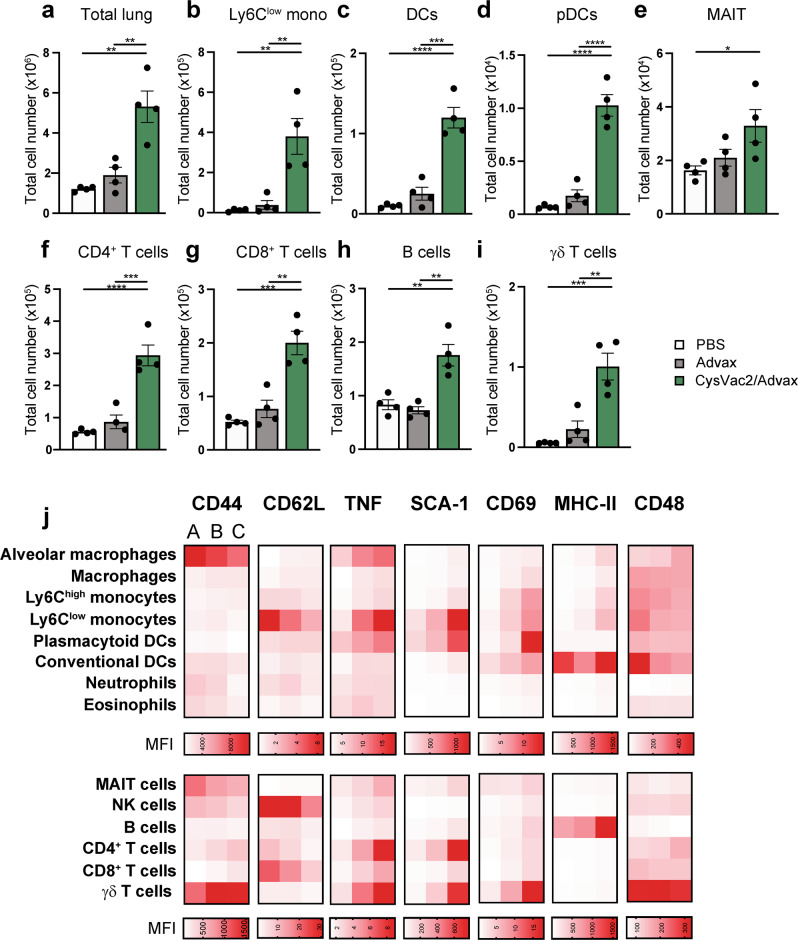
Intrapulmonary administration with Advax elicits innate and adaptive cell activation that is enhanced by presence of CysVac2 antigen. C57BL/6 mice were vaccinated i.t. with PBS, Advax-FITC or CysVac2/Advax-FITC and lungs and mediastinal lymph nodes were harvested for mass cytometric analysis 7 days later. Samples were treated with protein transport inhibitor cocktail (eBioscience) prior to fixation for intracellular staining. **a**–**i** Bar graphs depict the cell number of subsets ±SEM identified via CyTOF in the lungs of vaccinated animals. **j** Heat maps of geometric mean fluorescence intensity (MFI) expression of activation markers of (A) PBS, (B) Advax or (C) CysVac2/Advax vaccinated by lung cell subsets were generated using Prism 8 software, showing relative upregulation or downregulation in relation to PBS-treated group. Mean values represent *n* = 3–4 mice per group and are representative of two independent experiments. Significance of differences between groups was determined by ANOVA with post-hoc Tukey’s multiple comparison test (*p < 0.033, **p < 0.0021, ***p < 0.0002, ****p < 0.0001).

Given the relative lack of in-depth characterisation of pulmonary vaccines, we sought to gain a more detailed understanding of adjuvant/antigen driven phenotypic and cell population changes in the lung using mass cytometry. Mass cytometry allows the incorporation of a larger panel of markers without issues of spillover or compensation, thus allowing simultaneous identification of innate and adaptive cell subsets, including uncommon immune subsets (Figs. 6a and S3–6). The variety of immune subsets identified can be visualised based on multiple parameters using algorithms such as t-stochastic neighbour embedding (t-SNE), as depicted in Fig. 6a. At 7 days post-i.t. vaccination, CysVac2/Advax induced a significant increase in total cell number in the lung, compared to either adjuvant alone or PBS controls (Fig. 7a). This increase was reflected in an increase in various conventional innate subsets as well as unconventional lymphocytes such as mucosa associated invariant T (MAIT) cells (Fig. 7b–e), including those already observed via flow cytometry experiments (Figure S3a–f). Significant increases in adaptive immune subsets such as CD4^+^ T cells, CD8^+^ T cells, γδ T cells and B cells were also observed (Fig. 7f–i).

The activation status of the cell subsets identified was assessed by presentation of the geometric mean fluorescence intensity (MFI) of activation markers as a heatmap, assessing the expression of a range of activation markers (Fig. 7j) or by visualising the fluorescence intensity of specific markers expressed on individual cells in a t-SNE representation (Fig. 6b–d). Of note, upregulation of MHC-II compared to PBS was observed in the Advax alone group, and to a greater extent in the CysVac2/Advax group. This upregulation was particularly distinct in professional antigen presenting cell subsets such as macrophages, monocytes and DCs (Figs. 6b and 7j). TNF, responsible for broad activation of immune cells, was also upregulated across macrophage and monocyte subsets (Figs. 6c and 7j, upper panel). Furthermore, lymphocytes such as CD4^+^ and γδ T cells from CysVac2/Advax or Advax alone vaccinated mice displayed greater mean intensity of CD44, CD69 and TNF and lower levels of l-selectin than PBS vaccinated mice, reflective of an activated state. Also of interest, the mean intensity of the GPI-anchored protein, SCA-1, was markedly upregulated in CD4, γδ T cells and Ly6C low monocytes (Figs. 6d and 7j lower panel); this marker has been shown to be upregulated upon T cell activation and cytokine stimulation.^24^ Similar changes were observed in the mLN, including an increase in total cell number (Fig. S4a) and recruitment of myeloid subsets (Fig. S4b–g). The total number of B and CD4^+^ T cells in the mLN were also significantly increased after CysVac2/Advax administration (Fig. S4h, i); these subsets also displayed increased intensity of activation markers CD44 and CD48 on CD4^+^ T cells, and CD69 and MHC-II on T cells (Fig. S4l, m). Taken together, these results indicate that pulmonary CysVac2/Advax vaccination promotes enhanced accumulation of APC subsets in the lung-draining lymph node compared to adjuvant alone or PBS vaccination, which also coincides with improved activation and expansion of adaptive lymphoid cells.

## Discussion

Respiratory pathogens including *M. tuberculosis* significantly contribute to global morbidity and mortality, necessitating the development of more effective vaccines. Of increasing interest is pulmonary delivery as a novel vaccine strategy, as the protection generated by this delivery method can surpass that afforded by parenteral vaccination.^25^ In this study, we used fluorescently-conjugated forms of adjuvant (Advax) and antigen (CysVac2) to determine lung distribution and localisation of individual vaccine components. We observed that after intrapulmonary delivery, CysVac2 antigen is distributed in the tissue in close proximity to Advax adjuvant particles (Fig. 1) and that both vaccine components are distributed throughout the entire lung lobe (Fig. 2). Coupled with extracellular co-dissemination of vaccine components, we also observed colocalization of both adjuvant and antigen in a number of cells, both in the lungs and when administered to BMDCs (Fig. S1). It is possible that the magnitude of Advax particles delivered compared to CysVac2 antigen may increase the likelihood of cellular uptake of both vaccine components compared to antigen alone. It has been previously identified that antigen adsorption may not be required for Advax to potentiate its adjuvant effects, however there have not been any studies to specifically address the binding of antigen to the surface of Advax particles and further research into adjuvant-antigen interactions is required.^26^

In the delicate lung tissue, excessive granulocytic infiltration can cause damage, while conversely a lack of inflammation can generate tolerogenic responses. In this study, i.t. vaccination with CysVac2/Advax stimulated a transient influx of neutrophils, monocytes and eosinophils that abated one week after vaccine administration (Fig. 3). In the CysVac2/Advax vaccinated animals there was a significant increase in CD64^+^ macrophages compared to PBS controls, peaking at day 7 and returning to naïve levels at 3 weeks post-vaccination (Fig. 3c). There was also significantly higher eosinophil recruitment at day 3 post-vaccination in the CysVac2/Advax group compared to PBS vaccinated, that also returned to baseline levels by 7 days (Fig. 3h). Overall, analysis of lungs by mass cytometry at this time point reflected vaccine-induced changes observed by flow cytometry, with increased total cell number and number of macrophages, monocytes and neutrophils in CysVac2/Advax vaccinated mice compared to PBS controls, and no significant difference in eosinophil numbers at 7 days between groups (Figs. 7 and S3). There was an initial cytokine burst in the lungs observed at 6 h post vaccination, which may be responsible for early cellular recruitment (Fig. 4). At 7 days however there was a secondary increase in chemotactic factors, which could be driven by early recruited DCs, macrophages, monocytes and eosinophils. This chemotactic response may be responsible for the secondary peak in neutrophils observed at 7 days in both flow and mass cytometric analysis (Figs. 3g and S3d).

The varied recruitment of immune cells to the lungs, which did not appear to display strong Th1 or Th2 polarisation, was also paired with extensive adjuvant uptake (Fig. 3a–h). At early time points neutrophils appeared to be responsible for the most adjuvant uptake, reflective of their role as rapid responders to inflammation and as early transporters of antigen to draining lymph nodes (Fig. 3i).^27^ At 24 h, there was an increase in mononuclear phagocytic uptake of Advax particles, which was sustained over the timepoints measured. This may be a reflection of the size of Advax particles (2 µm in diameter) being highly amenable to macrophage phagocytosis.^28^ Moreover, the clear distinction of alveolar macrophages as the dominant Advax-positive subset at 22 days is likely reflective of their role in efferocytosis after acute vaccine-induced lung inflammation.^29^ This observation was also consistent with the substantial internalisation of Advax particles by CD11c^+^ cells observed via confocal imaging at this time point (Fig. 2).

Of note, we did observe Advax particles within the lung up to 22 days after i.t. vaccination (Fig. 2). The sustained presence of adjuvant following delivery has also been previously characterised for spray-dried H56/CAF01, which is present within the lungs 14 days following pulmonary vaccination,^30^ and Alum-adjuvanted vaccines which have detectable aluminium in tissue up to 9 months following subcutaneous or intramuscular delivery.^31^ Given the delicate nature of the respiratory mucosa, the persistence of adjuvant may be concerning in its potential to induce prolonged local inflammation. However, at 22 days total cell number and cytokine/chemokine concentrations had subsided to baseline levels (comparable to both PBS vaccinated and naïve mice), suggesting that the extended presence of adjuvant does not induce prolonged inflammation or cellular recruitment (Figs. 3 and 4). Confocal imaging at these extended time points revealed the majority of Advax to be cell-associated within CD11c^+^ positive subsets, with flow cytometry data suggesting these cells may be alveolar macrophages (Figs. 2 and 3i). It is possible that Advax particles resist degradation within the phagolysosomal compartments of alveolar macrophages, as a previous study has shown that Advax particles maintain their integrity in artificial lysosomal fluid for up to three weeks.^32^ As Advax is a form of inulin, it preferentially releases glucose and sucrose molecules when digested and is excreted in the urine when administered parenterally.^32^ After pulmonary administration, however, it is unclear how incomplete degradation of Advax particles may affect mucosal immune cell function, and this warrants further investigation.

Intrapulmonary vaccination with CysVac2/Advax induced a chemotactic response that also appeared highly regulated, subsiding by 10 days after vaccination (Fig. 4). These chemokines are known to recruit mononuclear phagocytes and CD4^+^ T cells to the lungs, and have been associated with attraction of Th2 helper subsets associated with allergic inflammation.^33^ However, we did not see prolonged eosinophilia or production of type 2 cytokines following intrapulmonary vaccination, such as that induced by alum adjuvant.^34^ This could be attributed to the propensity for chemokines, such as CCL3 and CCL17, to also perform a regulatory role in lung inflammation by limiting accumulation of granulocytes.^35,36^ As a polysaccharide adjuvant, it is possible that lectin receptors may recognise Advax and stimulate a combination of inflammatory and regulatory signals within cells, as lectin receptors are sometimes paired to inhibitory tyrosine-linked inhibitory motifs.^37^ It would be of interest to explore the immune subsets or mechanisms responsible for immunoregulation and if these responses are specific to Advax adjuvant.

While the mechanisms of action of adjuvants may differ, all are reliant on immune cell recruitment to the dLN following vaccination to promote the priming of adaptive immunity. The early increase at 6 h in neutrophil number in the mediastinal LN (Fig. 5) in CysVac2/Advax immunised mice is in line with previous studies observing rapid neutrophil antigen transport after parenteral vaccination.^27^ While neutrophils are well characterised as phagocytes and antigen transporters, their ability to present antigen and induce adaptive immune responses is not well established; however, they are known to enhance the initiation of the adaptive immune response in early *M. tuberculosis* infection.^38^ Monocytes and macrophages represented the largest number of adjuvant carrying cells arriving at the draining LN, perhaps reflective of the large size of Advax particles.^28^ While both cDC1 and cDC2 subsets observed in the LN are migratory subsets, cDC2 cells display a greater propensity for the activation of CD4^+^ subsets and promotion of CD4^+^ T cell homing to lung parenchyma, whereas cDC1 cells are known for better cross presentation of phagocytosed antigen to CD8^+^ T cells.^39^ The recruitment of both DC subsets to the draining LN and the increase in multiple lymphoid subsets after vaccination, suggests Advax adjuvant does not promote a highly polarised immune response. While our findings suggest a key role for migratory DC subsets in the induction of the adaptive response to vaccination, we cannot exclude a role for resident DC subsets within the LN in promoting the development of vaccine-induced responses. Subcapsular sinus DCs have been shown to mediate the rapid induction of T cell responses to particulate antigen within the LN; examination of their potential role in promoting vaccine-induced immunity is necessary to help guide vaccination efforts that target relevant APC subsets.^40^

Application of mass cytometry provided a detailed picture of vaccine-induced changes in the lung, including the activation status of recruited innate cells (Figs. 6 and 7). The activation of professional APCs and monocytes was characterised by increased expression of MHC-II, CD69 and TNF and was enhanced by the presence of antigen, in line with the observations of previous studies.^19^ Coupled with APC activation in the lungs we also observed increases in lymphocyte number and activation marker expression. Notably, we saw a prominent upregulation of SCA-1 on CD4^+^ and γδ T cells upon addition of CysVac2 to Advax (Figs. 6 and 7). SCA-1 is upregulated in response to TCR stimulation and cytokine expression, and is highly expressed on CD4^+^ T cell subsets with an effector memory phenotype (CD44^hi^ CD62L^low^).^24^ There is also evidence for SCA-1 as a regulator of cytokine-induced T cell proliferation, and this could feasibly contribute to immunoregulatory mechanisms that limit excessive cellular accumulation in the lung.^41^ Intrapulmonary CysVac2/Advax induced a prominent increase in the number of γδ T and MAIT cells, and augmented expression of the activation markers TNF, SCA1 and CD69 (Fig. 6). γδ T cells are a major source of cytokines, such as TNF and IL-17, at mucosal surfaces following infection; these may act to recruit or activate APCs or modulate the effector function of CD4^+^ subsets, thus contributing to protection against respiratory pathogens.^42^ This cell subset can also negatively regulate the development of Th2 responses and IgE production following antigen inhalation, which may provide an explanation for the limited eosinophilic response we observed after vaccination.^43^ MAIT cells, which are also mucosa associated, have been suggested as a key cell subset involved in vaccine-induced protection owing to their production of Th1 and Th17 cytokines.^44^ Accumulation and activation of pDCs was also apparent; while this subset is typically associated with TI IFN response, recent work suggests that activated pDCs can induce the production of a diverse range of cytokines, which may contribute to IFN-independent host defence against other pathogens.^45^ The observation that unconventional lymphocytes were also recruited to the mLN of vaccinated animals (Fig. S4) indicates further investigation is required to understand the importance of these cell types in the initiation of mucosal vaccine immune responses.

In conclusion, we have demonstrated that pulmonary delivery of CysVac2/Advax has extensive but transient immunomodulatory effects upon leucocytes in the lung and mLN. We determined that the pulmonary immune cell influx induced by Advax is not heavily polarised but instead involves a broad range of subsets, including unconventional immune cells that may contribute to vaccine-induced immunity. This study also contributes to the understanding of inflammatory signatures of pulmonary vaccination that may be important for generating lasting immunity against difficult respiratory pathogens such as *M. tuberculosis*. These data establish the potential of Advax as a mucosal subunit adjuvant for clinical translation in a range of vaccine platforms.

## Materials and methods

### Mice and vaccination

Female C57BL/6 mice 6–8 weeks of age were purchased from Animal Resources Centre (Perth, Western Australia). Mice were maintained in specific-pathogen free conditions and all mouse experiments approved by the Sydney Local Health District Animal Ethics Committee (protocol 2017-011). Construction of the CysVac2 fusion protein consisting of Ag85B and CysD was performed as described previously^22^ and was conjugated to AF647 using the Alexa Fluor™ 647 Protein Labeling Kit (Invitrogen, ThermoFisher Scientific, NSW, Australia) as per the manufacturer’s instructions. Fluorescein-conjugated Advax adjuvant (delta-inulin, 50 mg/ml) was supplied by Vaxine Pty Ltd (Adelaide, Australia). For vaccinations, mice were anaesthetised by intraperitoneal injection with ketamine (80 mg/kg) and xylazine (10 mg/kg) in PBS and vaccinated intratracheally (i.t.) with 1 mg Advax-Fluorescein/3 μg CysVac2 (AF647 conjugated or unconjugated) in PBS, 1 mg Advax-fluorescein in PBS or PBS alone using a PennCentury Microsprayer Aerosoliser (PennCentury, PA, USA).

### Assays of cytokine and chemokine production

Supernatant was taken from lung single cell suspensions prepared for flow cytometry of mice i.t. vaccinated with Advax-CysVac2, PBS or naïve mice and stored at −80 °C until use. Cytokine bead array (BD Biosciences, NSW, Australia) was performed on supernatant from single cell suspensions as per the manufacturer’s instructions. A standard curve was prepared by performing a 2-fold dilution series on cytokine standards of known concentration. Capture beads used were specific to: G-CSF, GM-CSF, IFN-γ, IL-1ß, IL-2, IL-4, IL-6, IL-10, IL-12/IL-23p40, IL-17A, IL-21 and TNF. The LEGENDplex Proinflammatory Chemokine Assay (Australian Biosearch, WA, Australia) was also performed on supernatant samples as per the manufacturer’s instructions. Capture beads used were specific to: CCL5, CCL20, CCL11, CCL17, CXCL1, CCL2, CXCL9, CXCL10, CCL3, CCL4, CXCL13, CXCL5 and CCL22. All multiplex assay samples were analysed on a LSR Fortessa X-20 cytometer.

### Preparation of samples for flow cytometry

Following euthanasia by CO_2_ exposure, lungs were perfused with chilled phosphate buffered saline (PBS) and then mechanically dissociated using a GentleMACS dissociator (Miltenyi Biotec, NSW, Australia). Lungs and mediastinal lymph nodes were then incubated with 10 U/mL DNAse I and Collagenase IV (Sigma-Aldrich, NSW, Australia) for 20 min at 37 °C before dissociation through a 70-µm cell strainer, washing and resuspension in RPMI/10% FCS for antibody staining. Single cell suspensions were resuspended in FACS wash (2% FCS, 5 mM EDTA in PBS) and incubated for 30 min with a mixture of fluorochrome labelled monoclonal antibodies detailed in Supplementary Table 1, Fixable Blue Dead Cell stain (Life Technologies, Thermo Fisher Scientific, NSW, Australia), and anti-CD16/32 blocking antibody (clone 2.4G2). Samples were acquired on a BD LSR-II (BD) and analyzed using FlowJo™ analysis software (Treestar, USA).

### Preparation of samples for mass cytometry

Antibodies were validated, pre-titered and supplied in pre-test amounts by the Ramaciotti Facility for Human Systems Biology Mass Cytometry Reagent Bank (University of Sydney, NSW, Australia). All steps until fixation were performed in the presence of Protein Transport Inhibitor Cocktail (Invitrogen, Thermo Fisher Scientific, NSW, Australia). Single cell suspensions were stained as previously described.^46^ Cells were incubated in 5 μM cisplatin (Fluidigm, NSW, Australia) for 5 min, then quenched with FACS wash and resuspended in surface stain monoclonal antibody mix listed in Supplementary Table 2. Following staining, cells were fixed in 4% paraformaldehyde then permeabilised using the eBioscience™ Foxp3/ Transcription Factor Staining Buffer Set (eBiosciences, Thermo Fisher Scientific, NSW, Australia). Cells were then stained with antibodies, washed and stored overnight in 4% paraformaldehyde with 0.125 µM iridium DNA intercalator (Fluidigm, NSW, Australia). For analysis, cells were washed in FACS wash, resuspended to a final concentration of 10^6^ cells/mL and samples acquired on a Helios Mass Cytometer (Fluidigm, NSW, Australia).

### Acquisition and analysis of mass cytometry data

EQ four element beads (Fluidigm, NSW, Australia) were added to samples immediately prior to acquisition for normalisation and analysis was performed using FlowJo v10.2. Sample cleanup was performed by gating on cells (DNA^+^), singlets and CD45^+^ cells, and then randomly down-sampled to reduce the total number of events to 10,000 per sample. Dimensionality reduction using t-distributed stochastic nearest neighbour embedding (tSNE) was performed using the FlowJo tSNE plugin, and population identities were confirmed using manual gating strategies (Figs. S6 and S7). Following tSNE analysis, the merged sample was separated into individual replicates and representative tSNE graphs showing fluorescence intensity expression were generated using R software.^47^

### Immunohistology

Mice were vaccinated via the i.t. route with 1 mg Advax-Fluorescein/3 μg CysVac2 (unconjugated or conjugated to AF647) as previously described, and euthanized at 1, 3 and 22 days post-vaccination. Lungs were prepared for imaging as described previously.^48^ mLNs were isolated from the same mice, transferred straight into 4% paraformaldehyde and processed for cryotome cutting as previously described. Sections were cut to 20 µm thickness for immunohistology and stained with anti-CD11c (clone 145-2C11, Biolegend, Australian Biosearch, NSW, Australia), anti-B220 (clone RA3-6B2, BD Biosciences, NSW, Australia), anti-CD3 (clone 145-2C11, BD Biosciences, NSW, Australia) and NucBlue Live ReadyProbes Reagent (Invitrogen, Thermo Fisher, NSW, Australia).

### Statistical analysis

Two-way or one-way analysis of variance (ANOVA) was performed where appropriate, with the Sidak post-hoc test used for comparison of multi-grouped data sets. Differences between groups were considered statistically different when *p* ≤ 0.05.

## Supplementary information

Supplementary Figures and Tables
