# Supplementary material for: Intrapulmonary vaccination with delta-inulin adjuvant stimulates non-polarised chemotactic signalling and diverse cellular interaction
Source: Mucosal Immunol. 2021 Feb 4;14(3):762–73. doi: 10.1038/s41385-021-00379-6 (PMC7859722; doi:10.1038/s41385-021-00379-6)
Supplement: Supplementary file 1 — Supplementary Figures and Tables [file 41385_2021_379_MOESM1_ESM.pdf]

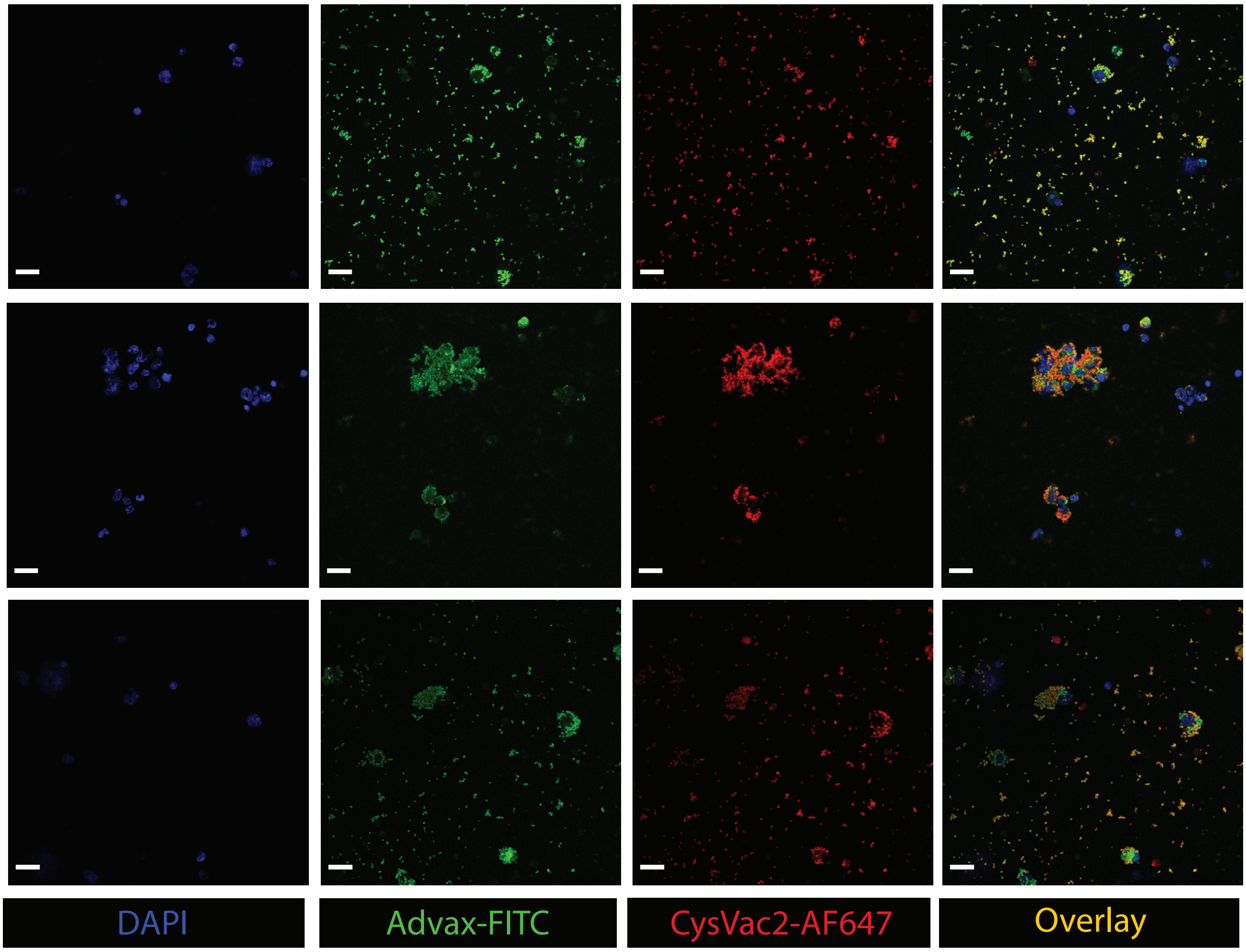

Supplementary figure 1: In vitro colocalisation of vaccine components incubated with bone marrow derived dendritic cells (BMDCs). Bone marrow was extracted from the diaphysis and epiphysis of a naive C57BL/6 mouse and established in culture with 20 ng/mL GM-CSF in complete RPMI media. Cells were differentiated over 7 days with GM-CSF replenished after 3-4 days. To examine colocalisation of vaccine components, 3 x 10^5 cells per well were incubated with 50ug Advax-FITC/ 5 ug CysVac2-AF647 for four hours. Culture components were streaked onto a microscopy slide and fixed with 4% PFA prior to staining with DAPI and imaging. Scale bars represent 20 microns.

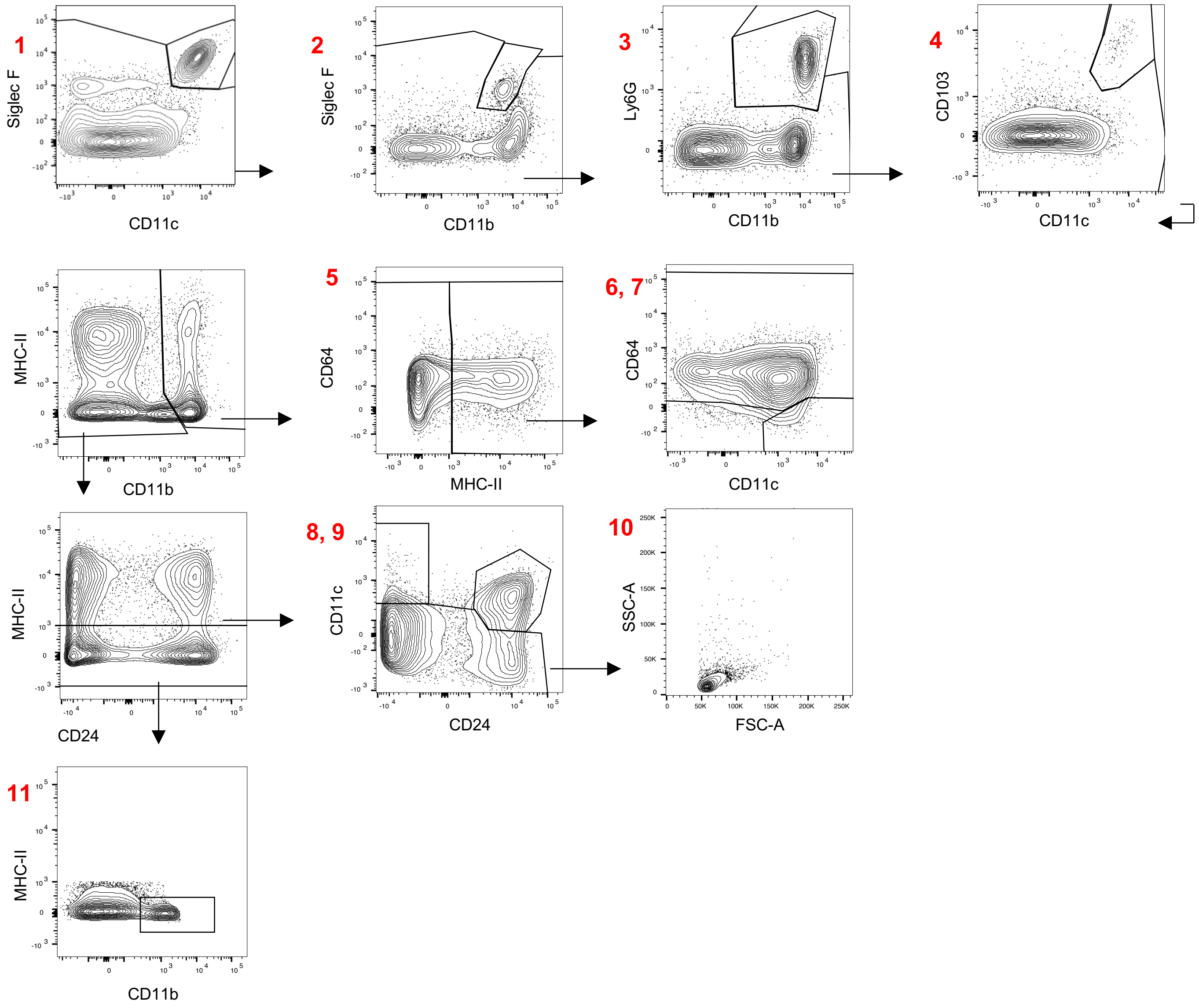

Supplementary figure 2: Flow cytometry gating strategy applied to lung and mLN samples

(1) Alveolar macrophages and (2) eosinophils were identified based on Siglec-F, CD11c and CD11b expression. (3) Neutrophils were identified as Ly6G^{+} and (4) CDC1 cells differentiated by their CD103 expression. (5) monocytes were distinguished by low MHC-II expression, while MHC-II^{high} populations were separated into (6) CD64+ macrophages identified as CD11b^{high}, MHCII^{+} and CD64^{+} and (7) cDC2 populations as CD11c^{high} CD64^{low}. (8) DC-like 1 cells or (9) DC-like 2 cells were identified as CD11b$^{low\text{-}mid}$, MHC-II^{+}, CD11c^{+} and CD24^{-} or CD24^{+} respectively. (10) B cells were identified based on absence of CD11c and CD11b, and by size and granularity. (11) Finall, NK cells were identified as CD11b^{mid} and MHCII^{-}

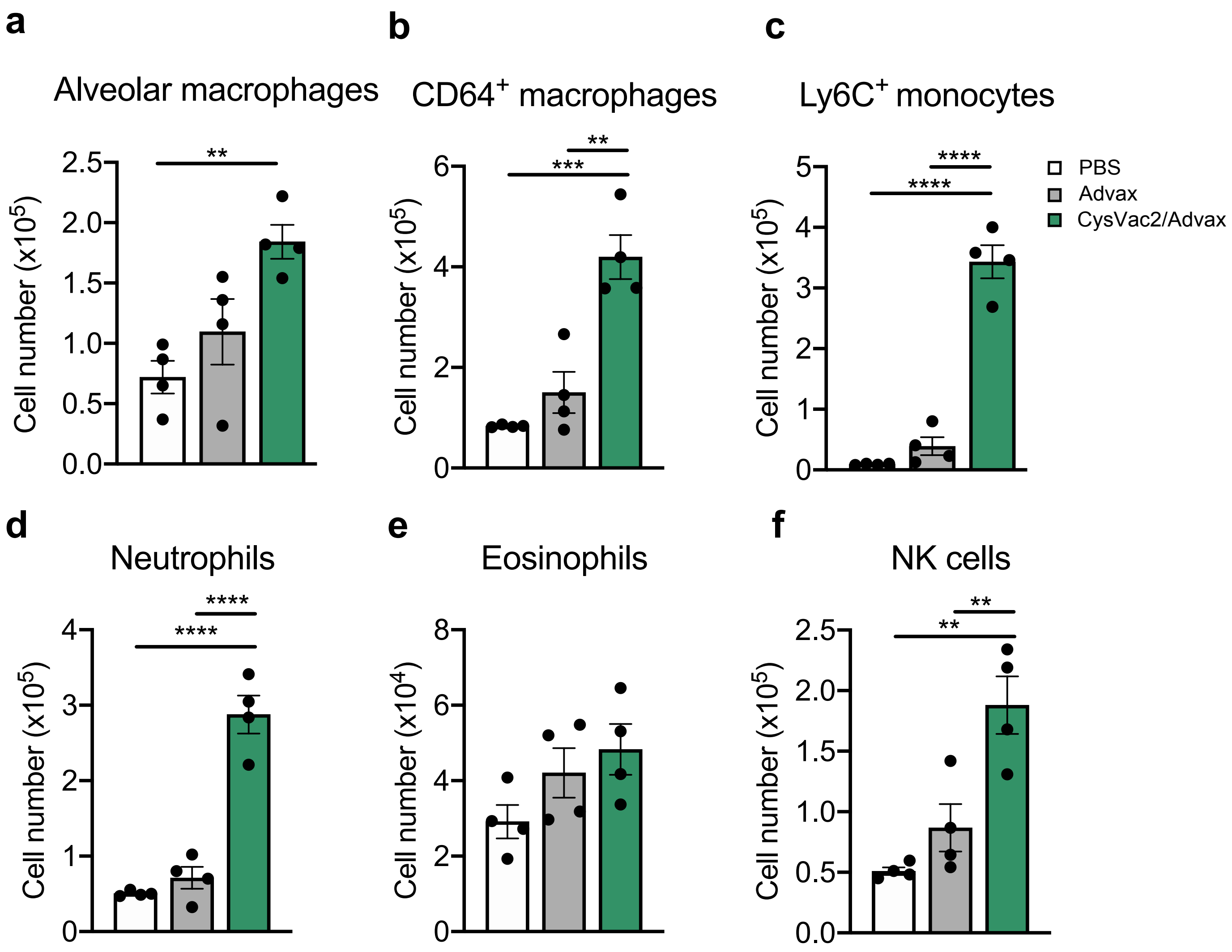

Supplementary Figure 3. **Intrapulmonary vaccination with Advax induces increased numbers of innate subsets in the lung which is augmented by addition of antigen.** C57BL/6 mice were vaccinated i.t. with PBS, Advax-FITC or CysVac2/Advax-FITC and lungs were harvested for mass cytometric analysis 7 days later. (a-f) Cell number of innate subsets identified via CyTOF in the lung of vaccinated animals. Mean values represent n=3-4 mice per group and are represen- tative of 2 independent experiments. Significance of differences between groups was determined by ANOVA with post-hoc Tukey's multiple comparison test (*p<0.033, **p<0.0021, ***p<0.0002, ****p<0.0001).

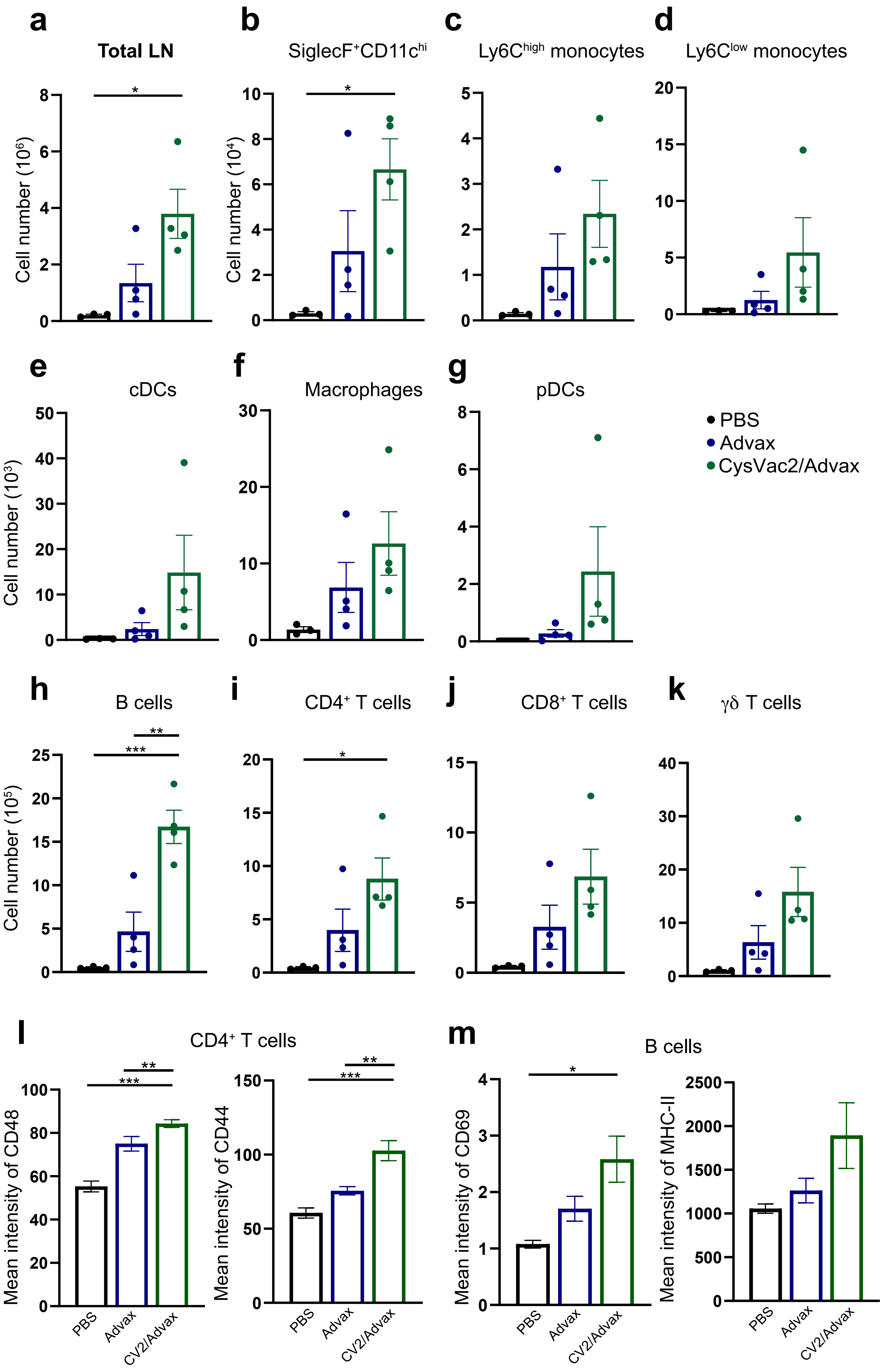

Supplementary Figure 4. Intrapulmonary vaccination with Advax induces recruitment and activation of innate and adaptive subsets to the lung-draining lymph node that is augmented with the addition of antigen. C57BL/6 mice were vaccinated i.t. with PBS, Advax-FITC or CysVac2/Advax-FITC and mediastinal lymph nodes were harvested for mass cytometric analysis 7 days later. (a-k) Cell number of innate and adaptive subsets identified via CyTOF in the mLN of vaccinated animals. (l, m) Mean ±SEM intensity of activation markers measured in the mLN by mass cytometry. Mean values represent n=3-4 mice per group and are representative of 2 independent experiments. Significance of differences between groups was determined by ANOVA with post-hoc Tukey's multiple comparison test (*p<0.033, **p<0.0021, ***p<0.0002, ****p<0.0001).

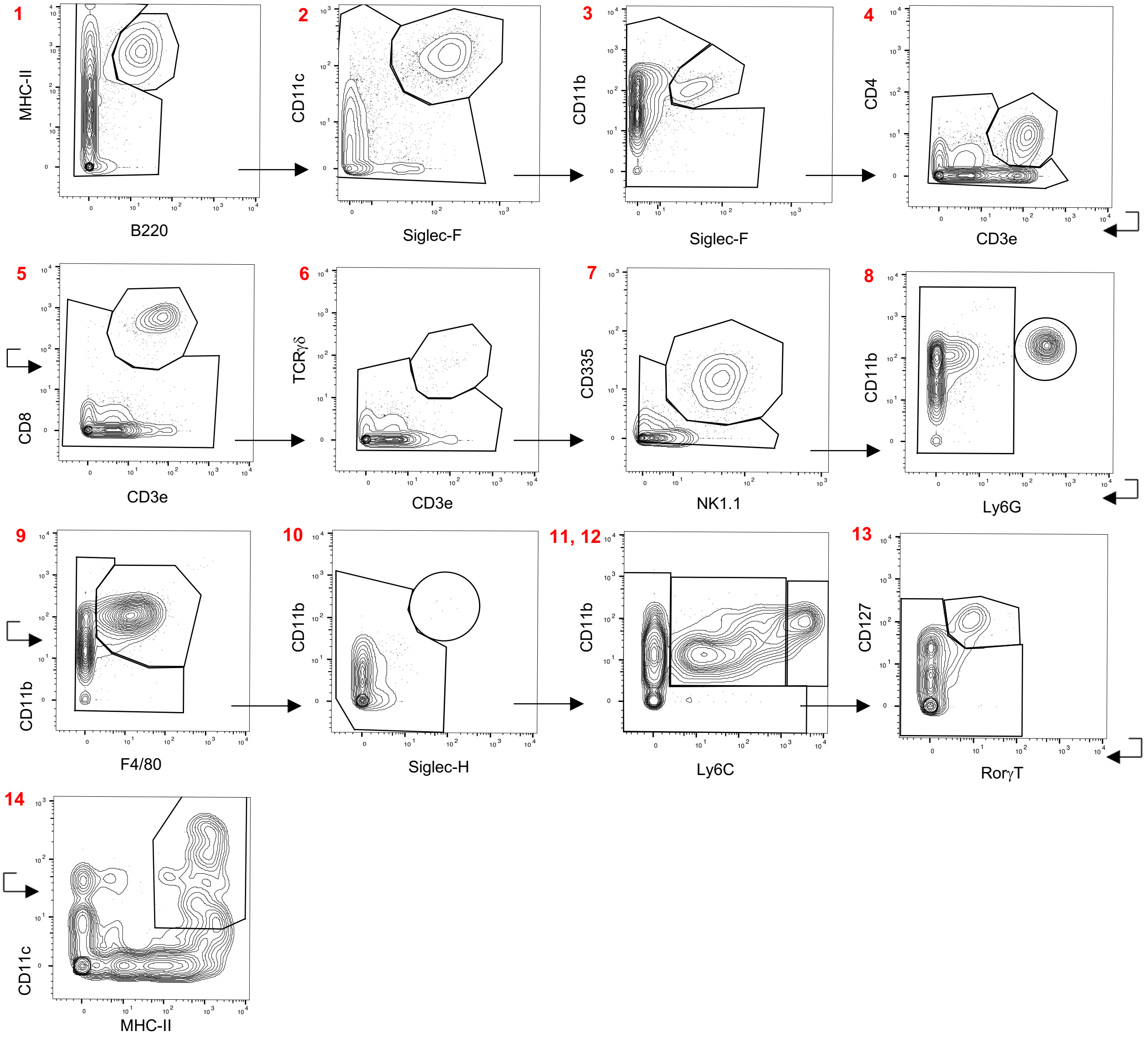

Supplementary figure 5: Manual gating strategy used to confirm tSNE grouping of innate immune subsets determined by mass cytometry. (1) B cells were identified as B220^{+} MHC-II^{+}. (2) Alveolar macrophages and (3) eosinophils were separated by Siglec-F expression and CD11c or CD11b respectively.T cells were identified as CD3e and (4) CD4^{+}, (5) CD8^{+} T or (6) TCR $\gamma\delta^{+}$ (7) NK cells were defined by CD446 and NK1.1 expression, and (8) neutrophils were separated using their Ly6G expression. (9) Macrophages were defined as F4/80^{+} and CD11b^{+}, and (10) plasmacytoid dendritic cells were separated based on Siglec-H expression. Monocytes were defined as (11) Ly6C mid or (12) Ly6C high. (13) MAIT cells were defined as CD127^{+} and RorγT^{+} and finally (14) classical dendritic cells were identified by their high MHC-II expression and CD11c^{+}.

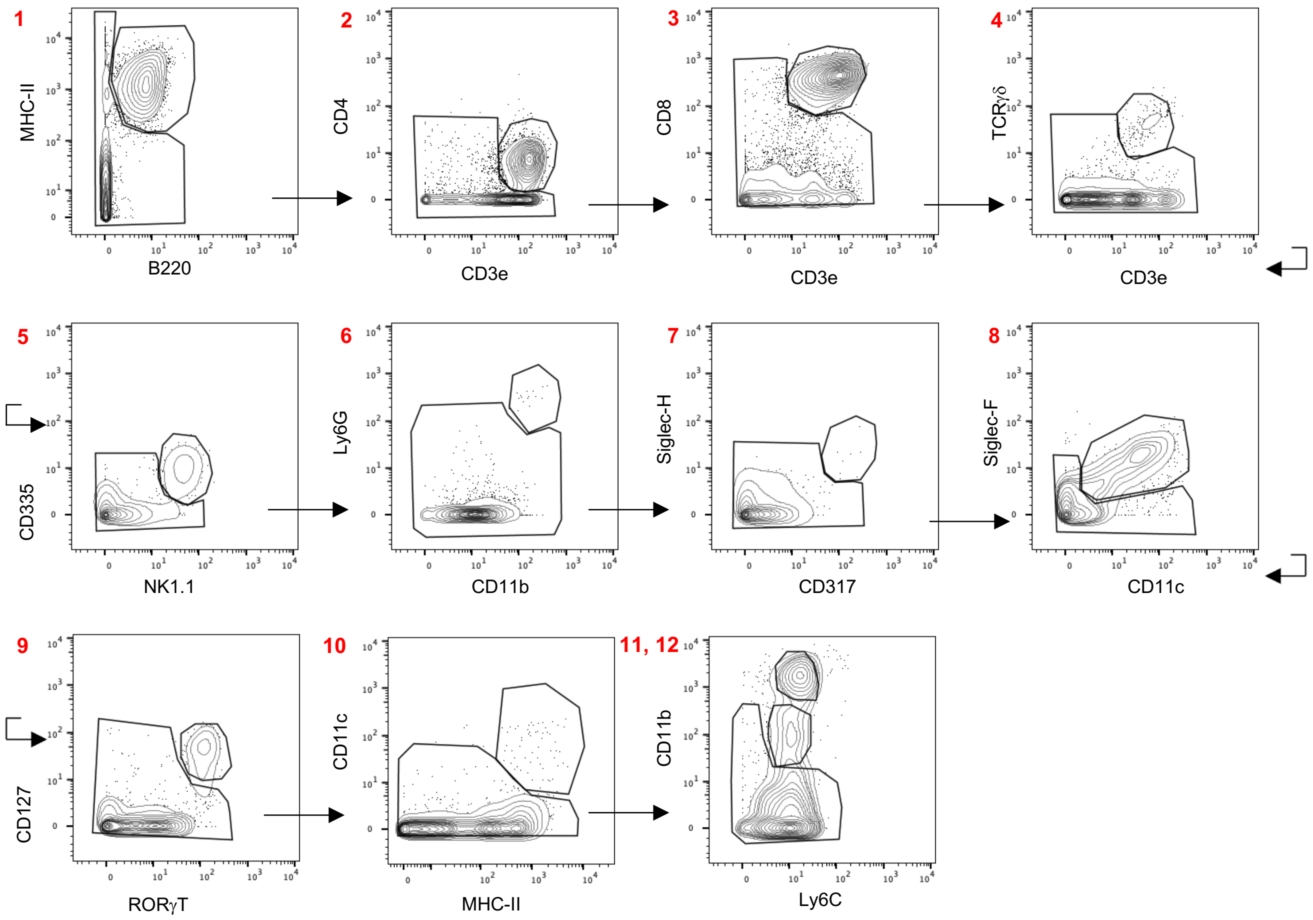

Supplementary figure 6: Mass cytometric gating strategy for identification of innate subsets in the mLN. After cleanup gating, cell subsets were identified as follows: 1) B cells were identified by their expression of B220, 2) CD4+ and 3) CD8+ T cells based on co-expression with CD3e. 4) Gamma delta T cells were identified by TCR-gd expression and 5) NK cells were defined by CD335 and NK1.1 expression. 6) Neutrophils were defined by Ly6G expression and 7) pDCs by high Siglec-H expression. 8) Siglec-F+ CD11c+ cells were identified based on co-expression of these markers 9) MAIT cells were identified as RORgT+ CD127+. 10) Conventional DCs were identified as CD11c and MHC-II high, and 11) Ly6Cmid and Ly6Chigh monocytes based on co-expression with CD11b.

Supplementary table 1: Antibodies used in flow cytometry

Antibody	Source
Anti-mouse Ly6G-BUV395 (clone 1A8)	BD Biosciences
Anti-mouse CD24-BUV737 (clone M1/69)	BD Horizon
Anti-mouse CD11c-AF700 (clone N418)	BioLegend
Anti-mouse CD11b-APC-Cy7 (clone M1/70)	BD Biosciences
Anti-mouse SiglecF-PE (clone E50-2440/E502440)	BD Pharmingen
Anti-mouse CD64-PE-Cy7 (clone X54-5/7.1)	BioLegend
Anti-mouse IA/IE-BV421 (clone M5/114.5.2)	BD Biosciences
Anti-mouse CD45.2-BV510 (clone 104)	BD Biosciences
Anti-mouse CD103-BV786 (clone M2-90/M290)	BD Horizon

Supplementary table 2: Antibodies used in mass cytometry

Antibody specificity	Metal label
Anti-mouse B220	89Di
Anti-mouse CD4	106Pd
Anti-mouse CD115	115Ln
Anti-mouse Ly6G	141Pr
Anti-mouse CD11c	142Nd
Anti-mouse FcER1a	143Nd
Anti-FITC	144Nd
Anti-mouse CD69	145Nd
Anti-mouse Siglec-F	146Nd
Anti-mouse CD45	147Sm
Anti-mouse CD11b	148Nd
Anti-mouse CD19	149Sm
Anti-mouse I-A/I-E	150Nd
Anti-mouse F4/80	151Eu
Anti-mouse CD3e	152Sm
Anti-mouse NKp46	153Eu
Anti-mouse CD169	154Sm
Anti-mouse CD80	155Gd
Anti-mouse CD48	156Gd
Anti-mouse CD317	158Gd
Anti-mouse TCRgd	159Tb
Anti-mouse CD62L	160Gd
Anti-mouse Siglec-H	161Dy
Anti-mouse TNFa	162Dy
Anti-mouse SCA-1	164Dy
Anti-mouse IFN-γ	165Ho
Anti-mouse IL-4	166Er
Anti-mouse IL-6	167Er
Anti-mouse CD8a	168Er
Anti-mouse RORγT	169Tm
Anti-mouse NK1.1	170Er
Anti-mouse CD44	171Yb
Anti-mouse Cy5	172Yb
Anti-mouse CD127	173Yb
Anti-mouse IL-17A	174Yb
Anti-mouse Ly6C	176Yb
Anti-mouse T-bet	209Bi
